# Candidate gene analysis using genomic quantitative PCR: identification of *ADAMTS13* large deletions in two patients with Upshaw-Schulman syndrome

**DOI:** 10.1002/mgg3.64

**Published:** 2014-01-14

**Authors:** Yuka Eura, Koichi Kokame, Toshiro Takafuta, Ryojiro Tanaka, Hikaru Kobayashi, Fumihiro Ishida, Shuichi Hisanaga, Masanori Matsumoto, Yoshihiro Fujimura, Toshiyuki Miyata

**Affiliations:** 1 Department of Molecular Pathogenesis, National Cerebral and Cardiovascular CenterSuita, Osaka, Japan; 2 Department of Hematology and Clinical Immunology, Nishi-Kobe Medical CenterKobe, Hyogo, Japan; 3 Department of Nephrology, Hyogo Prefectural Kobe Children's HospitalKobe, Hyogo, Japan; 4 Department of Hematology, Nagano Red Cross HospitalNagano, Japan; 5 Department of Biomedical Laboratory Sciences, Shinshu University School of MedicineMatsumoto, Nagano, Japan; 6 Department of Nephrology, Koga General HospitalMiyazaki, Japan; 7 Department of Blood Transfusion Medicine, Nara Medical UniversityKashihara, Nara, Japan

**Keywords:** *ADAMTS13*, genetic analysis, hereditary disease, mutation, quantitative PCR, thrombotic thrombocytopenic purpura, Upshaw-Schulman syndrome

## Abstract

Direct sequencing is a popular method to discover mutations in candidate genes responsible for hereditary diseases. A certain type of mutation, however, can be missed by the method. Here, we report a comprehensive genomic quantitative polymerase chain reaction (qPCR) to complement the weakness of direct sequencing. Upshaw-Schulman syndrome (USS) is a recessively inherited disease associated with severe deficiency of plasma ADAMTS13 activity. We previously analyzed *ADAMTS13* in 47 USS patients using direct sequencing, and 44 of them had either homozygous or compound heterozygous mutations. Then, we sought to reveal more extensive defects of *ADAMTS13* in the remaining three patients. We quantified copy numbers of each *ADAMTS13* exon in the patients by using genomic qPCR. Each primer pair was designed to contain at least one of the two primers used in direct sequencing, to avoid missing any exonic deletions. The qPCR demonstrated heterozygous loss of exons 7 and 8 in one patient and exon 27 in the other, and further analysis revealed c.746_987+373del1782 and c.3751_3892+587del729, respectively. Genomic qPCR provides an effective method for identifying extensive defects of the target genes.

Target exon resequencing using direct sequencing is a popular method to discover causative mutations in the candidate genes responsible for hereditary diseases. Homozygous or compound heterozygous mutations are often identified in the corresponding genes of the patients with autosomal-recessive diseases. In some cases, however, only one or no causative mutation is identified in the responsible gene: (an)other mutation(s) may be missed by the method. Although next-generation sequencing may be useful in such cases, it needs special equipments and is still expensive. In this study, we report a comprehensive genomic quantitative PCR (qPCR), which will be a powerful tool in combination with direct sequencing.

Upshaw-Schulman syndrome (USS), also called hereditary thrombotic thrombocytopenic purpura (TTP), is an autosomal-recessive trait associated with severely deficient plasma ADAMTS13 activity. Homozygous or compound heterozygous mutations in the *ADAMTS13* gene (OMIM 604134) are identified in most patients with USS (Levy et al. 2001; Kokame et al. 2002; Kokame and Miyata 2004; Matsumoto et al. 2004; Lotta et al. 2010; Fujimura et al. 2011; Hing et al. 2013). So far, more than 130 causative mutations have been identified by direct sequencing. Using that method, we previously analyzed *ADAMTS13* in 47 Japanese USS patients from 41 unrelated families (Fujimura et al. 2011). Of those, 44 patients from 38 families had either homozygous or compound heterozygous mutations in *ADAMTS13*. In the remaining three patients, however, only single missense mutations (two patients) or no mutation (one patient) was detected. In this study, we sought to reveal more extensive defects of *ADAMTS13* in these three patients by using genomic qPCR.

In general, PCR primer pairs for direct sequencing are designed to hybridize within the intronic sequences flanking each exon (Fig. 1A). Mutations such as substitutions, insertions, and deletions occurring in exons and exon–intron boundaries are identified by Sanger sequencing following genomic PCR, regardless of their heterozygosity or homozygosity (Fig. S1A). Direct sequencing, however, misses heterozygous mutations on the allele that contains no or mismatched primer target sequences: not only whole or partial deletion but also point mutations including single-nucleotide polymorphisms of primer target sequences can hamper PCR-amplification of the mutant allele, which may contain other critical mutations in the exon or exon–intron boundary (Fig. S1B). In these cases, only the target region of the other (normal) allele is PCR-amplified and sequenced, and the results are interpreted as if the regions of both alleles are normal.

**Figure 1:**
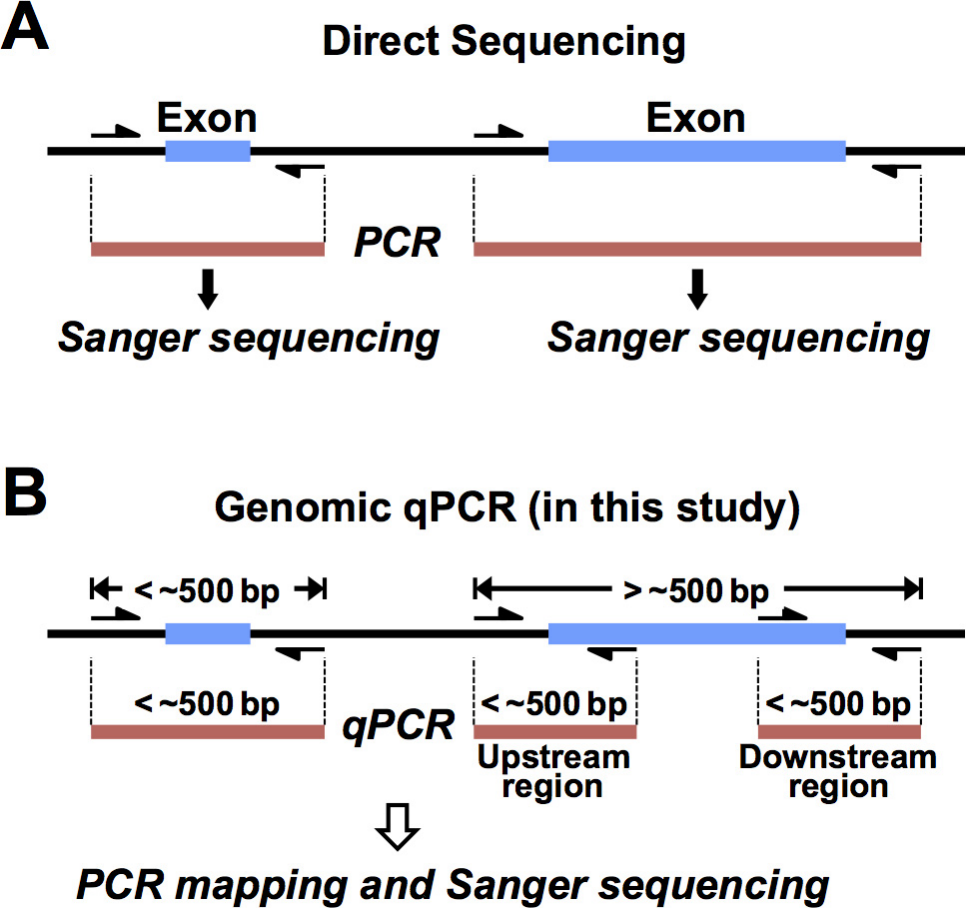
Principles of direct sequencing and genomic qPCR for genetic analysis. (A) In direct sequencing, target regions are amplified by PCR using primer pairs (arrows) usually designed from the intronic sequences flanking each exon, and the PCR products are directly sequenced by the Sanger method. (B) In genomic qPCR, copy numbers of target regions are quantified by real-time PCR. Each primer pair contains at least one of the two primers used in direct sequencing: common primer pairs are used for the regions smaller than ∼500 bp, and, for accurate qPCR, one common and one specific primer are used for the regions larger than ∼500 bp. If abnormal copy numbers are detected, PCR mapping and sequencing are performed to determine the precise sites of defects.

Copy number analysis may overcome the limitations of direct sequencing. Multiplex ligation-dependent probe amplification (MLPA) analysis (Schouten et al. 2002) is often used for this purpose. Although MLPA is suitable for detection of genetic defects including exon deletions and duplications, it may still miss mutations that occur outside the probe target sequences. Therefore, to complement direct sequencing, we selected genomic qPCR (Aldape et al. 2002; Kuramitsu et al. 2012), using primer pairs containing at least one of the two primers used in direct sequencing (Fig. 1B). Combining direct sequencing and genomic qPCR should reveal any defects occurring within or between primer target sequences.

The study protocol was approved by the ethical committee of the National Cerebral and Cardiovascular Center; only subjects who provided written informed consent for genetic analyses were included. This study involved three USS families, USS-W, -X, and -KK (Fig. 2A). Clinical data of the patients (USS-W4, -X5, and -KK3) and the basis of diagnosis were described previously (Fujimura et al. 2011). Plasma ADAMTS13 activities for patients and family members are shown in Figure 2A. No subjects had ADAMTS13 inhibitors. The results of direct sequencing are also shown in Figure 2A. USS-W4 was a heterozygote with paternal c.1648G>A (p.G550R), USS-X5 had no causative mutations, and USS-KK3 was a heterozygote with maternal c.841T>A (p.C281S). Pathologically unrelated missense polymorphisms (p.Q448E, p.P475S, p.G1181R) (Kokame et al. 2011) were also identified in them (Fig. 2A).

**Figure 2:**
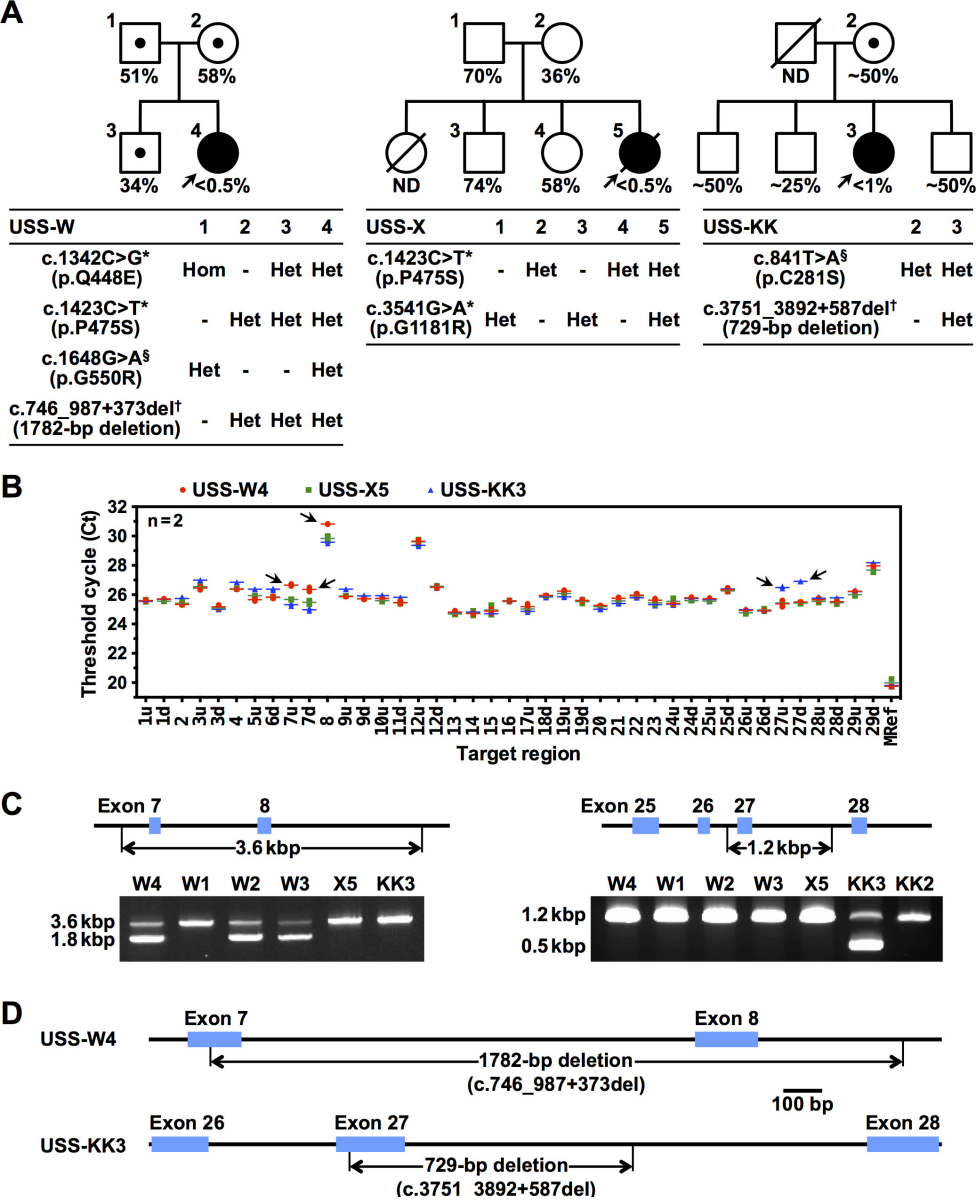
Genetic analysis of three USS families. (A) Pedigrees and genotypes of the USS patient families. Circles with arrows indicate the probands, USS-W4, -X5, and -KK3. Clinical data of the patients and the basis of diagnosis were described previously (Fujimura et al. 2011); the description of USS-KK3 being the second of three children needs to be corrected. Plasma ADAMTS13 activities were measured by us (USS-W and -X) or by Dr. Miha Furlan at University of Bern in 1999 (USS-KK), and are shown as a percentage of the normal control. ND, not determined. No subjects had ADAMTS13 inhibitors. Squares and circles with numbers indicate the subjects for genetic analysis. Each mutation was assigned a name for cDNA according to the nomenclature recommendations of the HGVS (http://www.hgvs.org/mutnomen/) based on the reference sequences AB069698.2 (cDNA) and NC_000009.11 (genomic). *^,§^Missense substitutions identified by direct sequencing. ^†^Deletions identified by genomic qPCR in this study. *Pathologically unrelated missense polymorphisms. (B) Identification of exon deletions in *ADAMTS13*. Ct values of genomic qPCR are plotted by dots with lines at the mean (*n* = 2) for each target region. The letters u and d following the exon numbers indicate *u*pstream and *d*ownstream region of each exon, respectively. Red circles, USS-W4; green squares, USS-X5; blue triangles, USS-KK3. Arrows indicate the dots with Ct values higher than those of the other two patients. (C) *Left*: PCR-amplification of the 3.6-kbp band from the normal *ADAMTS13* allele produced a 1.8-kbp band from USS-W4, her mother (W2) and her brother (W3), but not from her father (W1). *Right*: PCR-amplification of the 1.2-kbp band from the normal *ADAMTS13* allele produced a 0.5-kbp band from USS-KK3, but not from her mother (KK2). (D) Sequencing of the 1.8- and 0.5-kbp bands in (C) indicated a 1782-bp deletion in USS-W4 and a 729-bp deletion in USS-KK3, respectively.

Genomic DNA was prepared from blood and subjected to real-time PCR to quantify the copy numbers of each *ADAMTS13* exon. Each primer pair was designed, using Primer-BLAST (NCBI), to contain at least one of two primers used in direct sequencing (Table S1). A primer pair for the qBiomarker Multicopy Reference Copy Number Assay (MRef, Qiagen, Valencia, CA), which recognizes a stable sequence that appears >60 times throughout the human genome, was used to precisely normalize sample DNA input (∼4 ng/reaction). PCR was performed using the QuantiFast SYBR Green PCR Kit (Qiagen) for all regions except exon 7 and the KOD SYBR qPCR Mix (Toyobo, Osaka, Japan) for exon 7. Dimethyl sulfoxide was added (final concentration, 5%) for amplification of exon 8. Fluorescence intensities were detected using the Mx3000P QPCR System (Agilent Technologies, Santa Clara, CA), and each threshold cycle (Ct) value was calculated using the MxPro software (Agilent Technologies).

In genomic qPCR, the difference in Ct among subject DNAs is important information. An increase in Ct value of 1.0 indicates a heterozygous deletion of the target region, whereas a decrease of 0.58 indicates a heterozygous duplication. Ct values of the *ADAMTS13* qPCR indicated that exons 7 and 8 were heterozygously absent in USS-W4 and that exon 27 was heterozygously absent in USS-KK3 (Fig. 2B). By contrast, genomic qPCR revealed no abnormalities in USS-X5.

To confirm the deletions and narrow the deleted regions, we performed PCR using primer pairs specific to regions surrounding the deleted exons. Primers 5′-CACCTCCCCACAGACTCCTA-3′ (intron 6) and 5′-AGGCGGGCAAATCATGAGG-3′ (intron 8) amplified a 3.6-kbp band from the normal allele and a 1.8-kbp band from the mutant allele of USS-W4 (Fig. 2C, *left*). Thus, ∼1.8 kbp was deleted within the region straddling exons 7 and 8 in USS-W4. The precise sites where the deletions occurred were determined by sequencing the lower PCR band, which revealed that loss of exons 7 and 8 was caused by a 1782-bp deletion ranging from the 60th nucleotide of exon 7 to the 373rd nucleotide of intron 8 (c.746_987+373del1782) (Figs. 2D, S2A). We confirmed the compound heterozygosity of p.G550R and c.746_987+373del1782 in USS-W4 by genomic PCR of the family members. The patient's mother and brother, but not father, had c.746_987+373del1782 (Fig. 2C, *left*). Direct sequencing indicated that the patient's father, but not mother and brother, had p.G550R (Fig. 2A, *left*).

On the other hand, primers 5′-AGTCACATAGCCAGCAGTGG-3′ (intron 26) and 5′-GCACTGAGCAGAGTGGTCTT-3′ (intron 27) amplified a 1.2-kbp band from the normal allele and a 0.5-kbp band from the mutant allele of USS-KK3 (Fig. 2C, *right*). Thus, ∼0.7 kbp was deleted within the region straddling exon 27 in USS-KK3. Sequencing the lower band revealed that loss of exon 27 was caused by a 729-bp deletion ranging from the 36th nucleotide of exon 27 to the 587th nucleotide of intron 27 (c.3751_3892+587del729) (Figs. 2D, S2B). Although the patient's father could not be genetically analyzed, her mother had p.C281S (Fig. 2A, *right*), but not c.3751_3892+587del729 (Fig. 2C, *right*). Thus, it was likely that USS-KK3 was a compound heterozygote of p.C281S and c.3751_3892+587del729.

In conclusion, this study identified two USS patients carrying *ADAMTS13* alleles bearing exon deletions. Extensive defects of *ADAMTS13* may be more common than we expect, and genomic qPCR analysis will be effective for identifying such defects in USS patients. Of the three patients we examined, one did not exhibit abnormalities detectable by either direct sequencing or genomic qPCR. Because these combined analytical methods cannot detect large-scale events such as inversions and translocations that do not affect sequences or copy numbers of target regions, the patient may carry such a defect in *ADAMTS13*. Alternatively, plasma ADAMTS13 deficiency in the patient may be brought about by defects other than *ADAMTS13*, for example, genes involved in synthesis, folding, or secretion of ADAMTS13. Finally, we propose well-designed comprehensive genomic qPCR to complement the weakness of direct sequencing of candidate genes.

## Supporting Information

**Table S1.** Primer pairs for *ADAMTS13* genomic qPCR. **Figure S1.** Combinatorial analysis of direct sequencing and genomic qPCR should catch any defects occurring on and between the primer target sequences. Direct sequencing detects mutations such as point mutations (including substitutions, insertions, and deletions), short insertions, and deletions in the exons and exon–intron boundaries (A), but misses mutations on the allele that contains no or mismatched primer target sequences (B). Genomic qPCR for quantifying the copy numbers of target regions complements the results of direct sequencing. **Figure S2.** Deleted regions and flanking sequences of *ADAMTS13* identified in two patients with USS. The 1782- and 729-bp regions (red letters) were deleted in patients USS-W4 (A) and USS-KK3 (B), respectively. Lowercase and uppercase sequences indicate introns and exons, respectively. Underlined sequences adjacent to the breakpoints may cause microhomology-mediated end joining (MMEJ) (McVey and Lee. Trends Genet. 2008;24:529–538).
