# Supplementary material for: Candidate gene analysis using genomic quantitative PCR: identification of ADAMTS13 large deletions in two patients with Upshaw-Schulman syndrome
Source: Mol Genet Genomic Med. 2014 Jan 14;2(3):240–4. doi: 10.1002/mgg3.64 (PMC4049364; doi:10.1002/mgg3.64)
Supplement: Supplementary file 1 [file mgg30002-0240-SD1.pdf]

Table S1 Primer pairs for *ADAMTS13* genomic qPCR

Exon number	Forward primer	Reverse primer	Product size (bp)
1u	gattgccaggccgtttgtgat*	CAGTATGGAATGGGACAGGC	356
1d	CATCTTAGAGCAAGGCCCAG	gcaaaccccaaagctgatgta*	432
2	cctcggtctccccaagtgtta*	gaaccctggcctggctggaac*	348
3u	ggtgggggtgacacgcaatgt*	gagagcaagcagtggaaagc	92
3d	catccttccaagacctgcca	ccaggggagggagggagaaga*	400
4	tgttttccttgcgttagttgg*	gaggatggagatgcgatgact*	382
5u	aacaaaccgaccgcagtcagc*	catcagaaagctcggctacC	216
6d	tctctcaccgagGTTTGACC	ggttcccctgtcctcacacct*	317
7u	gctggcgctgcggcactaggg*	TCCGAAGCCATCACGTGTC	184
7d	TGAGCCTGCTCAGgtagc	gttggacggaggggtgggttg*	159
8	actcctccgtcccgcctcctc*	gccctcccaggactagctaca*	477
9u	gtgcagagtgttggctgtgtc*	AGGGCCTGGCACATATCcta	137
9d	TGGATGGGACAGAATGTGGC	ctctgccccatactggtcctg*	138
10u	tgaggatgttgggggactctc*	gtacCTGGGGTTGTTGCACT	245
11d	tccctagttgaaggcagtgg	caaatgtgtcctggtgtgaac*	222
12u	tgaggccacacccacatcttg*	cctatgactctgccctgtcc	312
12d	tcggacagggcagagtcata	atgccagagcctgaaccactt*	76
13	atagaaacccttgccccagat*	atccttttccccagcaccact*	390
14	cagggctgcagagtcattgag*	gaagggtggcgaagtggaaga*	358
15	ctccctttgtctgtggtgtgg*	actatcaagcctgagggtggt*	279
16	gggaccccgggaaggagagtc*	gtaagtgaccgctgaatgaat*	393
17u	gcttgctgaacgaaagattat*	CGTGGCTTAGGCTGGAAGT	201
18d	CAGGAAGGAGTTGGTGGAGA	cagtgtcctcacctgcagaat*	288
19u	accagcctgtgattcggttgt*	GGGGCAATGTCTTCAGGAG	298
19d	acgctctgtctccttcctca	aggaactctgacagcagcact*	381
20	ctctttgggctcctggatgtt*	caatgggtgctcctcgttctc*	386
21	aaggatacccgctgcgagacc*	agccaatcaacacccacattt*	489
22	ccatgcgggccttatgtgcta*	tctgggttgcagtcctcaaag*	439
23	gggggcctccagaaagagaac*	gtgttgcccaggttggacttg*	476
24u	ggctcagtggctgcactttcc*	AATGAGACAGGGGACACTGG	310
24d	agcctctctctggggtcttc	tccagcgtccccaaacctaag*	460
25u	gacagggacccagacttgaat*	CTGTGGTCCGTCCTGGAG	423
25d	tgggcaaaggcatcttcctc	aagttacttccccttgatagt*	146
26u	ctgcatgtgccccctcttgct*	CGCACTGCAGTTGAGAGAAC	374
26d	AGCCAACAGGAACCATTGAC	tgggcacatcacttaatctct*	322
27u	gtgcattcccacctgtagttt*	gcctggccatacCTCTGTAG	423
27d	GAGGAAGATGTGCAGGAAGC	tccctggcacgtgcagactga*	315
28u	ccagagcccagaacatttagc*	gggaaagctgtccagaatca	434
28d	AGTCCAGCCACGAGTAATGC	gccactatttcactcttgtag*	327
29u	gtgtccttggggaagtgatgt*	CCTCAGGTTCCTTCCTTTCC	397
29d	ACTGGGAGTCAGAGAGCAGC	gattggattttcttcctggat*	514

"u" and "d" following the exon numbers indicate upstream and downstream region of each exon, respectively.
Lowercase and uppercase letters indicate sequences within introns and exons, respectively.
Primers with an asterisk were also used for direct sequencing.

Supporting Information

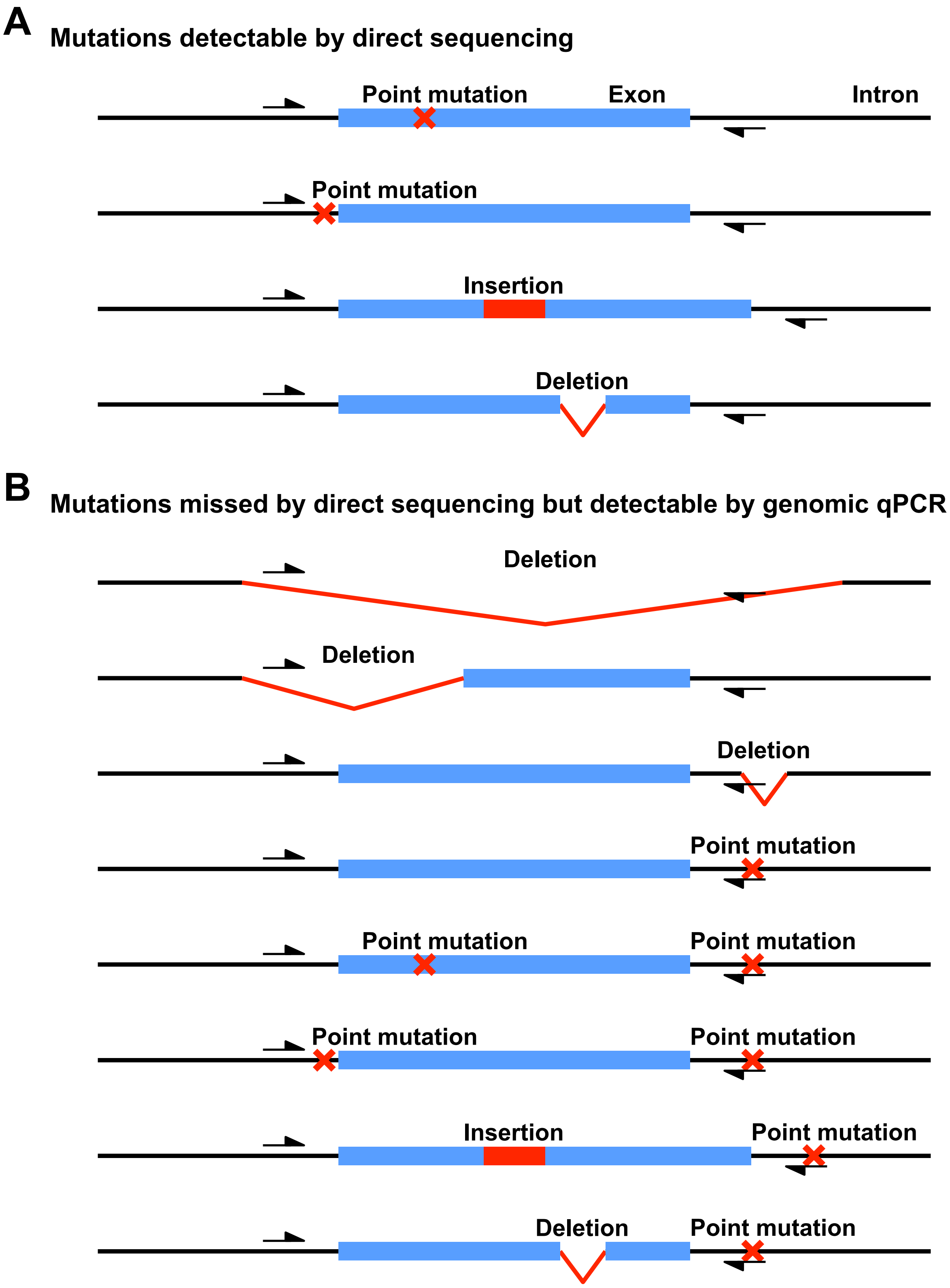

Figure S1. Combinatorial analysis of direct sequencing and genomic qPCR should catch any defects occurring on and between the primer target sequences. Direct sequencing detects mutations such as point mutations (including substitutions, insertions, and deletions), short insertions, and deletions in the exons and exon-intron boundaries (**A**), but misses mutations on the allele that contains no or mismatched primer target sequences (**B**). Genomic qPCR for quantifying the copy numbers of target regions complements the results of direct sequencing.

Supporting Information

A **c.746_987+373del: 1782-bp deletion identified in the USS-W4 patient**

c.687 ➡ Exon 7 c.746
ccccgaccagCTTCGGCCTGGAGCACGACGGCGCGCCCGGCAGCGGCTGCGGCCCCAGCGGACACGTGATGGCTTCGGACGGCGCCGCGCCCCGCGCCGG
Exon 7 ⬅ c.824
CCTCGCCTGGTCCCCCTGCAGCCGCCGGCAGCTGCTGAGCCTGCTCAGgtagcggccgccccgtgggaggggcgcgcgagcctccagccagcccgctggg
ccgccagcgccacctctctctacgtccgtccccactccgcattcagccctccttcctgtcccacccctccgtccaacccacccctccgtccaaccccgcg
cccaccgctccgtccgtggaggggcgggcgcgcgagcctccagccagcccgctgggccgcccgcgccacccctccctacgtccgtccccacctctcccta
cgtccgtccccactccgcattcagccctccttcctgtcctacctctccatcctgacccactcctccgtccaaccccgcgcccacagctccgtcccatccc
gctgcgcccactcctgcgcccacccctccgtcccaacccctgcacccacccccccgtcccacccacctgccccacccccctgcacctcccccgtgctgtc
ccactctgcggccacccttctgtccaacccctgcgcccaccgctccgttccacccccctccctgcgcccacccctgcgtcctccctcgcccccttgcgccc
acactttcgttccagccaatctgggcacgcacccctccgtccatccccatcccgccccttgactccacatacactccctggttctctcccacttgcctac
acccacccctgcatcctaccctcctccatccacccctccatctcagcccctgcacccaccccgttcctgggcccaccctgttcctgcacccaccccctc
acttcacccccttacccttcgtctgcctccacccgcccctacccctccgtccactctccacgctccatcagtcccacaccctatctcccccacccgcgt
acatgtatccctgcgtccccttcccgccgaccgcaccgctcccgggcctaacctgcatctgctccatcccactcagacccgtccctccgtcgccgctccc
tctgctggccacccacctctgcgccggcaggagccttagtcttggtcccagccaagagccggctcctggtggggggcgcgggccgagaactcctgttccc
actcacaaaggccacgcttccaaacgcttccatcctcgtgcccactcctccgtcccgcctcctcccggtgtacaccccgggactgagccgggcctgagc
c.825 ➡ Exon 8
cgggccttgtcgcagCGCAGGACGGGCGCGCTGCGTGTGGGACCCGCCGCGGCCTCAACCCGGGTCCGCGGGGCACCCGCCGGATGCGCAGCCTGGCCTC
Exon 8 ⬅ c.987
TACTACAGCGCCAACGAGCAGTGCCGCGTGGCCTTCGGCCCCAAGGCTGTCGCCTGCACCTTCGCCAGGGAGCACCTGGgtgagtctgccggcggtggcct
gggattggctgtgaggtccctccgcatcacccagctcacgtcccccaaaacgtgcatggtgagaacctgctgggtgccgtgctaggctgaggtactaagc
cagggcggcttagtttaatgctgtctgtgccctctagaaattatttaaaatgtttgaacaaaagctccaacattttgtttgactgggccccacaaatta
tgtagctagtcctgggagggcccctgtgcccaaggactcctggctgagtgaggacaccaatcttaaacagttaccaaggacttccccatctattgtggct
c.987+373
ggagtcagactggagggcttcctggaggaagtggcctctaaactgaacccacagcagaagtggggctggtagggggaggggagatgaaggagagcaggca

B **c.3751_3892+587del: 729-bp deletion identified in the USS-KK3 patient**

c.3716 ➡ Exon 27 c.3751
cgcttcctagGGGACATGTTGCTGCTTTGGGGCCGGCTCACCTGGAGGAAGATGTGCAGGAAGCTGTTGGACATGACTTTCAGCTCCAAGACCAACACGC
Exon 27 ⬅ c.3892
TGGTGGTGAGGCAGCGCTGCGGGCGGCCAGGAGGTGGGGTGCTGCTGCGGTATGGGAGCCAGCTTGCTCCTGAAACCTTCTACAGAGgtatggccaggcc
ttctccacctcccttgggtgctccagtcctggcagggaggctgggtgggtgctgctggggatggggccagtcccagtggggcagtgggaagatacggagg
gaactgactgagatggaaggaactggggttggccagtgtcagtctgcacgtgccagggaggggtcacaggatgaatgctatatccctccttttgggacc
gtgcagcaagatggacggatgtgggacatggtccacatcctcagtcagtccctcaggcctctgccccacacccacctgccccgcccccacccctccagcc
tttcaagggcttttagggttttgtggaagccactgtccctcagccctgtttcagtgcactggtgtaagcagacatgcttgtacatgcatgtgcacccaca
agcacacctcaggcagaggatgccacctcagggactccagccttgcccgtggcccccctcgatatcctctgatagccctctcggttgtcctgggggcttg
c.3892+587
ccctctcccaacagcccgagctggccgaagttggcttccctagctggttccagaggttcctcggctccccaggtgtctggggcttagtggcaacagggg

Figure S2. Deleted regions and flanking sequences of *ADAMTS13* identified in two patients with USS. The 1,782- and 729-bp regions (red letters) were deleted in patients USS-W4 (**A**) and USS-KK3 (**B**), respectively. Lowercase and uppercase sequences indicate introns and exons, respectively. Underlined sequences adjacent to the breakpoints may cause microhomology-mediated end joining (MMEJ) [McVey and Lee. Trends Genet. 2008; 24: 529–538].
